# Dr. Dalton’s Lectures

**Published:** 1852-03

**Authors:** 


*Dr. Dalton's Lectures in the University of Buffalo.* — The course by Dr. Dalton, on Physiology and Legal Medicine, in the University of Buffalo, terminated with the close of the session, having been continued during the whole of the preliminary and regular terms. As this is the first course of lectures given by Dr. D., it is but justice to him to say that the high expectations entertained by those most competent to appreciate his talents and acquirements, have been amply fulfilled. His success as a teacher has been all that could be desired by himself or his friends.

